# Aggressive pituitary tumors and pituitary carcinomas: Definition, management, and overview for clinical practice

**DOI:** 10.1093/noajnl/vdae114

**Published:** 2025-01-02

**Authors:** Francesco Calvanese, Gianpaolo Jannelli, Loic Feuvret, Alexandre Vasiljevic, Romain Manet, Camille Sergeant, Gerald Raverot, Emmanuel Jouanneau

**Affiliations:** Department of Neurosurgery, Helsinki University Central Hospital, Helsinki University, Helsinki, Finland; Skull Base and Pituitary Unit, Department of Neurosurgery B, Neurological Hospital Pierre-Wertheimer, Bron, France; Department of Neurosurgery, Neurocenter of Southern Switzerland, EOC, Lugano, Switzerland; Skull Base and Pituitary Unit, Department of Neurosurgery B, Neurological Hospital Pierre-Wertheimer, Bron, France; Radiation oncology Department, “Groupement Hospitalier Est,” Hospices Civils de Lyon, Bron, France; Inserm U1052, CNRS UMR5286, Cancer Research Center of Lyon, University Claude-Bernard Lyon 1, Lyon, France; Lyon 1 University, Villeurbanne, France; Histological Department, Reference Center for Rare Pituitary Diseases HYPO, “Groupement Hospitalier Est,” Hospices civils de Lyon, “Claude-Bernard” Lyon 1 University, Hôpital Louis-Pradel, Lyon, France; Skull Base and Pituitary Unit, Department of Neurosurgery B, Neurological Hospital Pierre-Wertheimer, Bron, France; Endocrinology Department, Reference Center for Rare Pituitary Diseases HYPO, “Groupement Hospitalier Est,” Hospices civils de Lyon, “Claude-Bernard” Lyon 1 University, Hôpital Louis-Pradel, Lyon, France; Endocrinology Department, Reference Center for Rare Pituitary Diseases HYPO, “Groupement Hospitalier Est,” Hospices civils de Lyon, “Claude-Bernard” Lyon 1 University, Hôpital Louis-Pradel, Lyon, France; Inserm U1052, CNRS UMR5286, Cancer Research Center of Lyon, University Claude-Bernard Lyon 1, Lyon, France; Lyon 1 University, Villeurbanne, France; Endocrinology Department, Reference Center for Rare Pituitary Diseases HYPO, “Groupement Hospitalier Est,” Hospices civils de Lyon, “Claude-Bernard” Lyon 1 University, Hôpital Louis-Pradel, Lyon, France; Inserm U1052, CNRS UMR5286, Cancer Research Center of Lyon, University Claude-Bernard Lyon 1, Lyon, France; Skull Base and Pituitary Unit, Department of Neurosurgery B, Neurological Hospital Pierre-Wertheimer, Bron, France

**Keywords:** adenoma, carcinoma, oncology, radiotherapy, temozolomide

## Abstract

**Background:**

Aggressive pituitary tumors and pituitary Carcinomas (PCs) represent very uncommon entities within the field of pituitary diseases. Unfortunately, treatment options after progression on temozolomide are limited. However, advances in the understanding of pituitary tumor genetics and their immunological landscape are paving the way for new targeted molecular therapies.

**Methods:**

In this article, we present an overview of the most recent literature, focusing on the specificities and role of current treatments and future perspectives in the management of these lesions.

**Results and Conclusions:**

Aggressive pituitary tumors and PCs remain very challenging conditions requiring a specific multidisciplinary approach in Pituitary Tumor Centers of excellence. If standard therapy fails, Temozolomide represents the first-line treatment option. Peptide Receptor Radionuclide Therapy may be also considered, especially in tumors expressing specific Somatostatin receptors. When tumors progress after Temozolomide treatment, the prognosis is typically poor, and among the various second-line treatment options immune checkpoint inhibitors have proven to be the most effective. Further studies exploring new potential targeted therapies and predictive factors for pituitary tumor aggressiveness are now essential to improve the management and outcomes for these patients.

**Key Points**

- Aggressive pituitary tumors represent rare entities.
- We believe that these tumors should be treated in Pituitary Tumor Centers of Excellence.
- A multidisciplinary approach including neurosurgeons, endocrinologists, oncologists, and radiotherapists is strongly suggested.

**Importance of the Study**

Our article focuses on the management of aggressive pituitary tumors and pituitary carcinomas. This represents a very complex topic, presenting significant challenges within the field of pituitary diseases. A review of the most relevant literature was performed, and we propose a paradigm for the management of these lesions. The importance of a multimodal approach is highlighted, including the experience of different specialists. We particularly point out the role of centers of reference for pituitary diseases in the management of these tumors. We hope that this article will improve our knowledge of these rare entities and contribute to providing new strategies for their diagnosis and treatment.

Pituitary adenomas (PAs) are overwhelmingly benign tumors arising from endocrine cells of the anterior pituitary gland. They are the third most common intracranial tumor in adults, representing 15% of these cases.^1,2^ Furthermore, PAs dominate the pituitary landscape, accounting for up to a staggering 94% of all pituitary lesions. However, the prevalence of clinically relevant PAs is approximately 80–100 cases per 100 000 individuals, with around 4 new cases per 100 000 individuals emerging each year. Consequently, there is growing concern about “incidentalomas,” meaning that we may be underestimating their true incidence.^3^

The clinical presentation and management of PAs relate both their close relationship with neurovascular structures of the sellar and parasellar regions and to the systemic alterations related to their hormone secretion. Because of this, these tumors should be treated in a Pituitary Tumor Center of Excellence (PTCOE) by a team including neurosurgeons, endocrinologists, pathologists, and radiation and medical oncologists who have both specific training and a scientific background.^4^

Most PAs are benign in nature and respond well to standard treatments including surgery, endocrine treatments, and radiation therapy. However, 2% of PAs (eg, an incidence of 1/18 000 in the general population) display aggressive local behavior, characterized by multiple recurrences and resistance to conventional treatments, leading to their classification as “aggressive pituitary tumors” (APTs) according to the 2018 European Society of Endocrinology (ESE) guidelines.^5^ Even more rarely, in about 0.2% of cases, pituitary tumors can metastasize systemically or in the cerebrospinal fluid, defining them as Pituitary Carcinomas (PCs).^6^ Based on these findings, the International Pituitary Pathology Group recently renamed PAs as “pituitary neuroendocrine tumors” (PitNETs), conferring a potentially oncological label to these otherwise benign entities.^7,8^ Even though this new denomination has not been universally accepted, it is undeniable that APTs/PCs defy the norms of “standard” PAs, presenting both a clinical course and outcomes that are wholly distinct and generally unpredictable at the time of diagnosis or at first treatment.^1,5,9^ Despite being infrequent in the general population, these tumors form part of daily clinical practice in PTCOEs. Unfortunately, when standard therapies fail only very few treatment options are available.^10^

Over the last decade, there has been a surge in both research and new oncological treatments to address these challenging cases, some of which remain off-label options. This has led to the increasing involvement of neuro-oncological members of the PTCOEs team.^11^

Our aim in this review is to shed light on and simplify the current definition, assessment, and treatment options available for APTs and PCs, particularly where conventional treatment protocols have proven ineffective.

## Diagnosis, Assessment, and Prediction

### Definition

PAs have recently been re-classified according to their immunohistochemical expression of specific transcription factors acquired by the anterior pituitary endocrine cells during embryonic development. In addition, in the 5th WHO classification, they have also been renamed PitNETs, giving them an oncological label. However, no staging or grading system has been proposed for this tumor due to their generally benign behavior.^9,12,13^

Although APTs and PCs are uncommon, they do occur and pose unique clinical and diagnostic challenges. Furthermore, the lack of clear definitions for these 2 entities has created several problems in estimating their epidemiology and identifying predictive markers.^6^

The first step is to distinguish APTs and PCs from invasive tumors. A significant proportion, up to 30%–40% of pituitary tumors, exhibits invasive behavior, infiltrating surrounding structures, as has been shown in major surgical series. Tumor invasion is usually assessed on MRI using the modified Knosp classification (Table 1) for extension into the cavernous sinus, even if distinguishing between simple extension and true invasion remains difficult.^14^

**Table 1.** Scheme of Knosp Modified Classification for Cavernous Sinus Infiltration by PAs

Grade	Criteria
Grade 0:	Tumor remains medial to the medial tangent.
Grade 1	Tumor extends to between the medial tangent and the intercarotid line.
Grade 2	Tumor extends to between the intercarotid line and the lateral tangent.
Grade 3a	Tumor extends lateral to the lateral tangent, above the intracavernous internal carotid artery into the superior cavernous sinus compartment.
Grade 3b	Tumor extends lateral to the lateral tangent, below the intracavernous internal carotid artery into the inferior cavernous sinus compartment.
Grade 4	Complete encasement of intracavernous internal carotid artery.

The 5th WHO classification does not include invasion as a predictive factor of aggressiveness.^8,15^ Indeed, even if APTs present as invasive macroadenomas, the 2018 ESE guidelines note that invasiveness alone is not synonymous with aggressiveness.

APTs are defined as radiologically invasive tumors showing unusually rapid tumor growth rate, or clinically relevant tumor growth despite optimal standard therapies (surgery, radiotherapy, and conventional medical treatments). On the other hand, PCs are currently distinguished from APTs solely by the presence of metastases (either in the cerebrospinal fluid or systemically).^5^

Histologically, there are no specific features that differentiate carcinomas from adenomas before metastasis occurs. Notably, an ESE survey published in 2016 on APTs and PCs revealed substantial clinical and histological similarities between the 2 groups.^16^ Trouillas et al. noted that APTs and PCs appear to be 2 variations of the same condition, and the former should be regarded as tumors with malignant potential.^12^ On the other hand, despite sharing some similarities, PCs more frequently exhibited a higher mitotic count (90% vs. 63%) and had a higher mortality rate when compared to APTs (43% vs. 28%) in the published cohort.^9^ However, the most recent ESE survey published in 2022 did not find any significant difference in mitotic count between PCs and APTs (number of tumors with >2/100 mitoses, APT: 31% vs. PC: 55%). Nevertheless, 44.8% of the 43 tumors graded as 2b (invasive and proliferative) and 55.1% of those graded as 2b* (invasive and highly proliferative) at initial surgery developed metastases. Similarly, the median survival from the time of first diagnosis was still later for APTs (17.2 years) compared to PCs (11.3 years), with a mortality risk which remained higher for PCs (HR, 1.58) even if this was lower after correction for secretion type, Ki-67 index >10%, and age.^17,18^ These findings confirm that APTs and PCs represent a continuum of the same entity and that evaluation of their proliferative status at diagnosis and at follow-up is mandatory (see below).

### Clinical Assessment

In the clinical realm, the definition of APTs requires further refinement, especially regarding the criteria used to define rapid tumor growth, clinically relevant growth, and resistance to standard treatments for functional pituitary tumors.

MRI is the gold-standard imaging method for pituitary tumors, providing crucial information about tumor location, size, and invasion into nearby structures.^6,16^ Comparing current imaging results with previous scans of the same tumor is of paramount importance to track tumor progression and to allow informed decisions about treatment strategies to be made. Imber et al. showed that a 1D approach, based on the Response Evaluation Criteria in Solid Tumors (RECIST) guideline 1.1, generally correlates with volumetric assessments, except for a few cases where a volumetric 3D assessment of progression is recommended (eg, multiloculated, multifocal and bony invasive tumors, small recurrences, or residual tumor).^19^ RECIST 1.1 considers a disease progressive when there is a 20% increase in the sum of the longest diameters of the tumor lesions, with a 5-mm absolute increase required to define progression.^6^ Furthermore, the appearance of new lesions can also signify progression.^20^

In the case of pituitary tumors, clinical aspects extend beyond mere absolute volumetric criteria. Indeed, clinical aggravation can be evident even in radiologically stable disease, especially when the tumor is close to critical structures, such as the optic chiasm or cranial nerve in the lateral wall of the cavernous sinuses.^1,21,22^

Identifying unusually rapid growth in pituitary tumors represents a challenge due to their typical slow progression and variability in the time interval between initial diagnosis and the onset of aggressive tumor behavior, which ranges from months to over a decade. A recent study has shown that up to half of 151 APTs/PCs studied were initially considered benign before later demonstrating aggressiveness.^17^ To identify this unusual growth pattern, it is necessary to compare the most recent imaging data not only to the previous images but also to historical scans. If the tumor exhibits a 20% increase in size within a period of less than 6 months (or less than 12 months if annual MRI scans are the only available option), it is considered to be unusually rapidly growing.^5,21^

### Histological, Molecular, and Genetic Predictive Factors of Aggressive Behavior

One of the most problematic issues in dealing with APTs/PCs is that they are only defined after progression, recurrence, and resistance to standard treatment. Therefore, a significant number of studies focus on finding clinical and molecular predictive factors of recurrence and aggressiveness. This approach aims to customize treatment and follow-up for each individual case from the start of management.

APT/PCs are usually secreting tumors, and a change in function of a previously nonfunctioning tumor should raise an alarm about a possible change in behavior towards tumor aggressiveness.^17^ In the 5th WHO classification, certain tumor types have been listed as high-risk or potentially aggressive tumors, including sparsely granulated somatotroph adenomas, lactotroph adenomas in men, Crooke’s cell adenomas, silent corticotroph adenomas, and immature plurihormonal Pit1-positive adenomas.^8^ However, these classifications require more evidence from prospective studies for validation.^7^

Histopathological evaluation, specifically immunohistochemistry for pituitary hormones, Ki-67 proliferation index, mitotic count, and p53 immunostaining, is fundamental for categorizing tumors with aggressive potential. It is recommended that Ki-67 index, p53 immunostaining, and mitotic count all be evaluated.^5,6^ Remarkably, in the last ESE survey a significant proportion of APTs/PCs in the survey had Ki-67 levels ≥10%, compared to only 3% in another series.^17^ At the same time, p53 mutations, previously considered rare in APTs/PCs, have been reported more frequently in particular cases that are characterized by recurrence after surgery. The actual number of APTs/PCs with p53 mutations may be higher and thus requires further research. Furthermore, a precise cutoff for immunocytochemistry of TP53 in pituitary tumors has not yet been established, unlike the case in other endocrine tumors such as gastroenteropancreatic carcinomas.^23^ However, it is essential to recognize that no single marker is sufficient on its own to predict tumor behavior, and a combination of markers provides a more comprehensive assessment.^9^

In 2013, a French 5-tiered clinicopathological classification for pituitary tumors was proposed, to guide the identification of more APTs at the time of diagnosis.^9^ This classification considers tumor diameter, type, and grading based on invasion and proliferation. Invasion is assessed by MRI and histopathology findings in the cavernous sinus and sphenoid sinus mucosae. Only tumors classified as grades III and IV of Knosp’s classification are considered as invasive. Proliferation is assessed using 3 parameters: mitotic count, Ki-67 labeling index, and p53 positivity. This classification categorizes tumors into 5 grades: Grade 1a (noninvasive and non-proliferative), grade 1b (noninvasive and proliferative), grade 2a (invasive and non-proliferative), grade 2b (invasive and proliferative), and grade 3 (metastatic tumor). Notably, grade 1a tumors are the most common, while grade 2b tumors represent a smaller percentage. The prognostic value of this classification has been validated in several studies, demonstrating its significance in predicting disease-free survival and risk of recurrence.^24^

Germline genetic testing is recommended in APTs, particularly when patients are young or have a family history of pituitary or endocrine neoplasia.^25^ Although APTs and CPs are more frequent in patients carrying germline mutations, these have not to date been associated with aggressive behavior.^5,6,26^Somatic mutations in genes such as ATRX and TP53 have shown promise in predicting tumor behavior. Seven haplotypic variants of SF3B1 may be associated with aggressive tumor behavior and poor clinical outcomes in prolactinomas. Additionally, some transcriptomic studies have highlighted a possible correlation between different genetic mutations in ADAMTS6, PTTG, CRMP1, ASK, AURKB, CENPE, and CCNB1 in lactotroph tumors, while other studies have shown that decreased RIZ1 (PRDM2) expression was associated with invasiveness and resistance to dopamine agonists^5^. It has also been reported that USP8 mutations are more frequent in small noninvasive ACTH-secreting tumors in men, but conclusive data are lacking. A considerable proportion (20%–30%) of studied cohorts of USP8 WT macroadenomas have shown a coexisting TP53 mutation and were characterized by recurrence after surgery and an aggressive clinical course.^1,27^ Finally, chromosomal instability and specific copy number variations also hold potential as prognostic markers.^1,21,23,27^

The tumor microenvironment, which includes immune cells, fibroblasts, cytokines, chemokines, and blood vessels, plays a crucial role in pituitary tumor aggressiveness.^10,26^ Notably, tumor-associated macrophages, in particular the M2-like subtype, appear to promote invasiveness and proliferation, while immune cells create an immunosuppressive microenvironment.^28^ Understanding the intricate crosstalk within the tumor microenvironment and the interplay of various markers offers insights into effectively managing APTs, albeit with the need for further research and validation of these markers.^6^ Combining these markers and understanding their interplay within the tumor microenvironment offers promising future avenues for the effective management of APTs/PCs.

## Resistance to Standard Treatment and the Need to Control Hypersecretion

### Failure of Medical Treatment

Resistance to standard treatment needs to be properly defined. Rapid relapse or progression after surgical removal as well as tumors that progress after radiotherapy (RT) represent classical clinical issues. In terms of medical treatment, APTs pose unique challenges. Standard therapies, that are often effective for non-aggressive tumors, exhibit limited success in aggressive cases.

### When Should We Suspect an APT?

In the case of prolactinomas (or PIT1-PitNETs), resistance to dopamine agonists is characterized by a failure to achieve normal prolactin levels and obtaining less than a 50% decrease in tumor size despite escalating doses of cabergoline, up to 3.5 mg/week. Taking into account that reduced expressions of dopamine D2 receptors and estrogen receptors have been proposed as possible causes of resistance, high doses of up to 11 mg/week have been advocated for normalizing prolactin levels.^29^ Therefore, it has been suggested that the highest tolerated dose of dopamine agonists should be administered to patients with aggressive prolactinomas, as this may overcome resistance. When this is not effective some studies have also used pasireotide with varying results.^30^ Otherwise, a potential APT should be evoked.

In acromegaly, treatment resistance is defined as the complete lack of biochemical and tumor response and occurs in less than 10% of patients. For aggressive somatotroph tumors, when medical treatments with somatostatin (SST) receptor ligands, growth hormone receptor antagonists, or dopamine agonists achieve partial control of hormone secretion, these can then be continued at the highest tolerated doses. Once again, there are no clear limits which evoke suspicion of an APT.^5,6,21^

For patients with hypercortisolism and APT/PCs, managing cortisol levels is vital since complications from cortisol excess could be fatal. Effective control of hormone hypersecretion is crucial, preferably through medical interventions such as steroidogenesis inhibitors and/or pasireotide, following a comprehensive approach involving surgery and RT. While bilateral adrenalectomy is an option, this carries the risk of promoting further tumor growth.^1,31,32^

A lack of control of hypersecretion or recurrence of hypersecretion despite a good volume response of the pituitary tumor indicates the need for screening for metastases, including brain and spine MRI, total body CT-scan, and/or PET/SPECT, and/or DOTATOC Scan. Standard medical treatments do not arrest the growth of aggressive gonadotroph/nonfunctioning PAs but fortunately, these are APTs or PCs in only a minority of cases.^12,17^

### Failure of Radiotherapy

Even though several months of treatment could be required to control hormone secretion, pituitary tumors usually respond well to RT regardless of tumor type.^1^ Moreover, it is generally accepted that the efficacy of anti-secretory treatment occurs more rapidly with Gamma Knife radiosurgery than with external beam radiation therapy.^33^ For this reason, the absence of response to adjuvant or upfront radiation therapy, as well as early recurrence after treatment, should evoke a potential APT/PC.^34^

## Treatment Options

Table 2 summarizes the response rate reported for the different medical therapies currently used as first- and second-line treatment in APT/PC in major clinical studies.

**Table 2.** Summary of Different Systemic Treatment Options for PCs and APTs

Authors	Therapy	Mechanism of action	Patients	Type of tumor	Outcome
McCormack et al., 2016	Temozolamide (9 cycles)	Oral alkylating agent that acts by DNA methylation.	156	116 APTs, 40 PCs,	Complete response in 6%, partial response in 31%, stable disease in 33%, and progressive disease in 30%.
Lizzul et al., 2020	Temozolamide (14–45 cycles)		8	7 APTs and 1 PC	No patient had progression disease during long-term treatment nor toxicities. No one had a complete response but 4 had a partial response.
Lsolle et al., 2017	Temozolamide (6,5 cycles)		43	29 APTs and 14 PCs	22 patients (51.2%) were considered as responders, with a significantly higher overall survival.
Ebel et al., 2020	Temozolamide (6 cycles)		47	34 APts and 13 PCs	Tumor regression in 20%, stable disease in 17%, and tumor progression in 63%. Progression occurred 16 months after initiation of TMZ.
Zacharia et al., 2014	Capecitabine and Temozolomide (CAPTEM)		4	4 APTs (functioning corticotroph)	Complete regression in 2 patients, 75% regression in one patient, and stable disease for >4.5 years in 1 patient.
Kovács et al., 2013	Peptide receptor radionuclide therapy	Radiolabelled peptides—mainly radiolabelled somatostatin receptor ligands -	1	APT (functioning corticotroph)	NA, but the patient died of elevated intracranial pressure shortly after the treatment.
Komor et al., 2014	Peptide receptor radionuclide therapy (177Lu-DOTATOC, 3 cycles)		1	APT (nonfunctioning)	Stable disease.
Maclean et al., 2014	Peptide receptor radionuclide therapy (177Lu-DOTATATE, 4 cycles)		3	2 APTs and 1 PC	Stable disease 40 months after completing the planned 4 cycles of treatment in one patient. Two patients with rapidly progressive atypical adenomas terminated treatment early due to continued disease. progression.
Waligórska-Stachura et al., 2016	Peptide receptor radionuclide therapy (90Y-DOTATATE, 4 cycles)		1	APT (Functioning somatotroph)	Tumor volume and hormonal response: Partial response at 12 months.
Raverot et al., 2022 (systematic review)	Immune checkpoint inhibitors	Anti PD-L1 Antibodies Anti CTLA4 antibodies	24	16 Functioning corticotroph (7 APTs and 9 PCs) and 8 functioning lactotrophs (4 APTs and 4 PCs)	Functioning corticotroph: 6 complete responses, 1 partial response, 2 stable diseases, 7 progression diseases. Functioning lactotroph: 2 partial responses, 1 stable disease, 5 progression diseases.
Raverot et al., 2021 (systematic review)	Bevacizumab	Anti-VEGF antibody	17	10 APTs and 7 PCs (6 functioning corticotroph, 2 functioning lactotroph, 1 functioning somatotroph, and 8 tumors of unknown subtype)	1 complete response, 4 partial response, 7 stable disease.
Raverot et al., 2021 (systematic review)	Tyrosine kinase inhibitors	Inhibition of EGFR, ALK, BCR–ABL and VEGFR	12	10 APTs and 2 PCs	1 partial response, 5 stable diseases, 6 progression disease.
Raverot et al., 2021 (systematic review)	Everolimus	PI3K–AKT–mTOR pathway inhibitors	7	4 APTs and 3 PCs (3 functioning corticotroph, 1 functioning lactotroph, 3 tumors of unknown subtype)	1 stable disease, 6 progression disease.

### The Role of Surgery and Radiotherapy

Conventional local treatments, such as surgery and RT, still play a primary role in the management of APTs/PCs.

With regard to surgery, it is strongly recommended that the surgery should be performed by highly experienced neurosurgeons due to the correlation between postoperative outcomes and surgeon experience.^4,35^ It is important to differentiate between recurrence following sub-optimal surgery and recurrence after surgery performed by an expert. Therefore, reevaluation by an expert surgeon in a PTCOE is the first step in management. Approximately 80% of patients with APTs and PCs undergo surgery at least twice, including 27.9% who have at least 4 surgeries.^5,6^ In most cases, complete tumor resection is not achievable, and the role of surgery is to provide surgical debulking to alleviate the local mass effect of clinically relevant parts of the tumor, to control hormone hypersecretion, and reduce or reshape the lesion to enhance the effects of further adjuvant treatments such as RT and chemotherapy. Surgery also allows histopathology to be updated, including molecular analysis, possibly indicating options for new therapeutic targets. Since APTs/PCs are life-threatening diseases, it may be necessary to perform more extensive surgery via endoscopic parasellar or transcranial approaches. However, the current literature does not provide any guidelines and the indication should thus be decided based on case-by-case discussions within a multidisciplinary team in a PTCOE.^22^

RT plays a crucial role in the management of APTs/PCs, especially when there is clinically relevant tumor growth despite surgery in nonfunctioning tumors, or after both surgery and standard medical treatment in functioning tumors.^6,33^ Both external beam radiation therapy and stereotactic radiosurgery are effective options. RT may offer long-term control of tumor growth, but this indication should be carefully considered, weighing the potential benefits against the risks of hypopituitarism and the rare but serious long-term side effects, including the increased risk of malignant brain tumors or optic pathway injury.^34^ In some cases, adjuvant RT may be recommended when there is a clinically relevant invasive tumor remnant showing pathological markers that predict aggressive behavior (eg, Ki-67 index, mitotic count, and p53 immunostaining).^36^ This decision should involve expert radiation oncologists who can assess the best RT options based on tumor size, location, prior radiation, and pathology.^37^ Special care must be taken in outlining the target for RT because some recurrences or failures of RT are due to a gross total volume which does not cover the whole tumor bed rather than to tumor aggressiveness. In PTCOEs, the target is usually established by the surgeon, the radiologist, and radiation oncologist using a multimodal approach, with the ideal tumor volume to be irradiated being the initial tumor volume and not the tumor remnant. For these reasons, stereotactic radiosurgery-Gamma Knife is probably the best approach for treating APTs.^4,34^

Re-irradiation alone, or associated with temozolomide (TMZ), has been shown to be effective for local control in patients with recurrent PAs, with an acceptable rate of toxicity including optic neuropathy, hypopituitarism, and medial temporal lobe necrosis.^38–40^ Nevertheless, re-irradiation is less effective than primary treatment in controlling hormonal hypersecretion and in the management of APTs/PCs this is not usually the primary aim compared to local control.^6,38^

### Temozolomide

TMZ is an oral alkylating agent known for its good tolerance and intracranial bioavailability. It exerts its effects by inducing DNA methylation, leading to irreversible DNA damage. The action of TMZ can be counteracted by O6-methylguanine-DNA methyltransferase (MGMT), a DNA repair enzyme responsible for removing added methyl groups.^6^ TMZ is typically administered at a dose of 150–200 mg/m^2^ daily in cycles of 5 days every 28 days and is recommended as first-line treatment for APTs/PCs when standard therapy fails.^5^ Positive effects on overall survival and progression-free 5 years survival have been found in patients who are TMZ-responders.^41,42^ For patients who have not previously received the maximal RT dose and subsequently experienced rapid tumor growth, the Stupp protocol is suggested.^43^ This protocol involves concurrent TMZ at 75 mg/m^2^ daily with RT, followed by TMZ alone at 150–200 mg/m^2^ daily, with treatment cycles lasting 5 days every 28 days. The recommended treatment duration is at least 6 months, with the potential for longer administration if the treatment continues to be effective.^16,21,22,30^

In a recent meta-analysis, Luo et al. analyzed the results of 21 studies involving 429 patients.^44^ The overall radiological and biochemical response rates to TMZ were found to be 41% and 53 %, respectively. The survival rates at 2 and 4 years were 79% and 61%, respectively, with a progression-free survival of 20.18 months and an overall survival of 40 months. The benefit of TMZ varied according to the functional tumor subtype of patients with greater antitumor activities found in subgroups with functional tumors, while concomitant chemoradiotherapy improved the radiological response rate to 60%.

Similarly, in a systematic review published by Raverot et al. in 2018, the response rate after the first course of TMZ was estimated to be 47% in 11 studies involving 106 patients (34 with carcinomas).^5^ In this cohort, only 6% achieved a complete response, 31% had a partial response, 33% showed stable disease, and 30% had progressive disease after the first course of TMZ. The first evaluation of response was carried out at 3 months and progression on TMZ at this point indicates resistance. It is worth noting that 25% of tumors that initially responded and 48% of those initially with stable disease progressed after stopping the first course of TMZ, indicating that the outcomes of a second course of TMZ are less effective. Positive predictive factors for TMZ response were functional tumors, concurrent RT, and low MGMT expression. While weak MGMT staining was generally associated with better treatment responses, this correlation is not always consistent, making TMZ an option even in patients with high MGMT levels.

Similar results have emerged from a more recent ESE multicenter survey, in which a response was observed in 37% of 156 out of 171 patients receiving first-line treatment with TMZ alone.^17^ Among responders, only 9.6% achieved a complete response, 30.1% had a partial response, 28.1% showed stable disease, and 32.2% had progressive disease after the first course of TMZ. The estimated mean duration of the effect of TMZ in responders was 6.4 and 3.3 years after achieving complete and partial responses, respectively, and 1.4 years in patients with stable disease. Among the 31 patients who received a second treatment with TMZ, only 5 out of 17 responders and 1 out of 11 with stable tumors maintained their sensitivity, while 4 out of the 11 patients with stable disease experienced a subsequent period of disease stabilization lasting at least 1 year. Considering the limited availability of alternative treatment options, it may be worth considering a second course of TMZ for patients who responded positively to the first round.

For tumors progressing after the first course of TMZ, treatment options are limited.^41^ Clinical trials are ongoing to assess the benefits of combination therapy (NCT03930771) and the Stupp protocol versus RT alone (NCT04244708). According to the 2022 ESE survey, 7 out of 9 patients with clinically functional tumors who received the Stupp protocol or RT within 6 weeks before stopping the first TMZ course showed a partial or complete response.^17^ In some limited cases, researchers have explored the combination of TMZ with other drugs, with capecitabine (the CAPTEM regimen) being the most common choice. However, the results so far have not shown significant improvements compared to TMZ monotherapy.^45^

Maximizing and prolonging the efficacy of TMZ is crucial. This may involve long-term administration of the drug as long as it remains effective and well-tolerated, or incorporating TMZ in the management of these tumors earlier, rather than solely as a “rescue therapy.”^46^

In a recent review, the rate of grades 2–4 TMZ-related adverse effects in patients treated for APT/PC was 19%.^44^ These included pancytopenia, hepatopathy, and minor side effects such as nausea, vomiting, loss of appetite, mouth sores, changes in taste, constipation, tiredness, dizziness, trouble sleeping, and headache. Thus, a team member with oncological expertise is crucial for monitoring and managing these issues.

### Immunotherapies

Immune checkpoint inhibitors (ICIs) are emerging as a novel therapeutic approach for APTs/PCs. These molecules block immune checkpoints, which are considered to be “brakes” on the immune system, and consequently reactivate and enhance antitumoral immune responses. Targets of these humanized antibodies are PD1/PD-L1 (antibodies include novolimubab and pembrolizumab), CTLA4/CD80-86 (ipilimimab), and LAG-3 (relatlimab), blocking tumor-induced downregulation of cytotoxic t-lymphocyte activity and, as a consequence, stimulating these cells to kill the cancerous cells.^45,47^

Several histopathology studies have reported the presence of a tumor-infiltrating lymphocyte population (T CD8+) in APT/PCs and the expression of programmed death-ligand 1 (PD-L1), which is considered a potential predictor of ICI response. Moreover, preclinical data from murine models of Cushing’s disease also point to the potential efficacy of these drugs in APT. Finally, one of the adverse effects reported with ICIs is autoimmune hypophysitis, suggesting an action of this agent on the pituitary. Another important aspect is that previous administration of conventional chemotherapy agents, such as TMZ, may enhance the effectiveness of ICIs due to the somatic hypermutation induced by chemotherapy.^6,48,49^

Since the first successfully treated case in 2018, the use of ICIs as second-line treatment in APTs/PCs has stimulated increasing interest. In a systematic review published in 2023, a total of 28 cases of APT and PC have been reported as being treated with these agents to date.^48^ Specifically, 15 cases belonged to a large cohort, 4 cases were from a small phase 2 trial, and there were 9 other isolated reports.^50–61^ Among these, all received ICIs as second-line regimes after multimodal treatment, which included TMZ in 27 cases. Nineteen cases were corticotroph tumors (11 PC and 8 APT), while 9 were Pit1-lineage, with 4 being PCs. Among the 19 corticotroph tumors, 1 (3.6%) and 6 (21.4%) showed a complete and partial clinico-radiological response, respectively, with a duration of between 3 and 42 months; 2 tumors remained stable for approximately 12 months while the remaining 10 (36%) progressed. Concerning lactotroph tumors, partial response and stability were observed in 3 (33.3%) and 1 (11.1%), while the remaining 5 tumors progressed regardless of the treatment (55.5%). PCs responded better than APTs to ICI treatments, especially the corticotroph tumors. Moreover, unlike lactotroph tumors, in corticotroph tumors negative PD-L1 staining and very low CD8 + T cell infiltration in the center of the tumor did not rule out ICI treatment. Finally, even if a high mutational burden and microsatellite instability in tumors seem to predict the response to treatment for all tumor types, further validation is required.

Despite being associated with a high rate of major adverse effects, combined treatment (ie, ipilimumab plus nivolumab ± followed by maintenance nivolumab) appeared to be more effective than monotherapy (ie, pembrolizumab or nivolumab followed by ipilimumab). Combined treatment led to a response in 7/19 patients (36.8%), stability in 3/19 (15.8%), and progression in 9/19 (47.3%), while monotherapy led to a response in 3/9 patients (33.3%) and progression in 6/9 (66.7%).^48,50,51,53^ For PCs, in the case of a dissociated response between the different metastatic lesions or between primary tumor and peripheral metastases, integrating a local treatment (ie, surgery, radiotherapy, and hepatic alcohol ablation) with ICIs for non-responding tumors appears to be a viable option. Taking into account the immunomodulatory effect of glucocorticoids, aiming for eucortisolism before starting ICI treatment appears prudent.^49^

All of these findings suggest a specific role for ICIs as second-line treatment after progression on TMZ or the Stupp protocol, especially for pituitary corticotroph carcinomas.^10,22,49,56^ Ongoing clinical trials are currently investigating combination therapy with nivolumab and ipilimumab (NCT02834013 and NCT04042753), which may clarify the role of ICIs in the treatment algorithm for APT/PCs.

Apart from ICIs, the realm of immunotherapy offers other intriguing possibilities for the treatment of APTs/PCs. These options include oncolytic viruses, cancer vaccines, and a focus on tumor-associated macrophages. While the effectiveness of these therapies in PCs and APTs remains uncertain, there is considerable interest in targeting tumor-associated macrophages due to their presence and significant roles in pituitary tumors. This approach may hold particular promise for gonadotroph tumors, which seem to be less responsive to ICIs compared to other histological types.^6,23^

### Bevacizumab

Bevacizumab is a humanized anti-vascular endothelial growth factor (VEGF) antibody used in oncology to inhibit tumor angiogenesis. Administration of bevacizumab in APT/PCs has been reported so far in 30 cases, of which at least 7 were carcinomas (11 corticotroph tumors, 8 lactotroph tumors, 2 somatotroph tumors, 2 tumors negative for pituitary hormones and 7 tumors of unknown subtype).^17,56,60,62–67^ Of these, only 18 cases were treated with Bevacizumab monotherapy and this mostly represented second-line chemotherapy after at least a first course of TMZ. Disease stabilization was achieved in 8 cases and a partial response in 1, with treatment duration which ranged from 6 to 26 months, while all other cases progressed, and the results were unavailable for 4 cases. Most of the remaining 12 cases received bevacizumab, combined with TMZ and/or RT, rendering the impact of bevacizumab on the outcome difficult to assess due to the known efficacy of these treatments on APT/PCs.^48^ Although adverse effects were not consistently reported, side effects were reported in some cases, including epistaxis, hypertension, and nephritis. These findings suggest that bevacizumab holds promise for the management of APTs, and it has thus been suggested as a second-line treatment.^16,17^ However, further research is needed to determine its long-term efficacy and safety.

### Other Molecular Targets

Various targeted therapies have been explored as potential treatments for APTs/PCs. Tyrosine kinase inhibitors are a class of drug that targets specific proteins involved in tumor growth, development, and angiogenesis. Among these, the epidermal growth factor receptor (EGFR) pathway has received significant attention for treating anterior pituitary tumors.^68–70^ However, results from tyrosine kinase inhibitor treatment have been variable. For example, the dual EGFR1/EGFR2 inhibitor, lapatinib, showed encouraging results in some cases of lactotroph tumors, achieving disease stabilization for 6 months in 3 out of 4 patients in a small pilot study.^71,72^ On the other hand, an EGFR1 inhibitor (erlotinib) had no effect on a corticotroph APT.^6^ Another promising avenue is sunitinib, which inhibits multiple tyrosine kinase receptors, including the VEGF receptor. However, it showed no effect in 3 cases of APTs.^17^ A combination of TMZ with apatinib, a VEGFR-2 inhibitor, led to marked regression in a single case of a somatotroph APT.^16,23^

Everolimus, an mTOR pathway inhibitor, has been used in a total of 8 cases of APTs/PCs (3 corticotroph, one lactotroph, and 4 undefined tumors). This molecule achieved disease stabilization, sustained for a year, in only 1 patient harboring a lactotroph APT, while all other cases showed inexorable progression. Additionally, a corticotroph PC carrying an mTOR pathway mutation showed no progression on a combination of everolimus and RT.^6,17^

Furthermore, palbociclib, a cyclin-dependent kinase 4/6 inhibitor, which targets a pathway that is frequently over-activated in PCs and recurrent tumors, has shown effectiveness in a non-aggressive, nonfunctioning pituitary tumor that was concurrent with metastatic breast cancer.^73^

Overall, these targeted therapies offer potential avenues for the treatment of APTs and PCs, with ongoing research now aimed at refining their use and identifying patient subgroups that might most benefit from these treatments.

### PRTT

Peptide Receptor Radionuclide Therapy (PRRT) is an emerging approach that uses radiolabeled peptides, primarily SST receptor ligands, to deliver precise radiation to tumors expressing the corresponding receptors. The rationale for utilizing PRRT in this context stems from the widespread presence of SST receptors in anterior pituitary tumors and their capacity to take up these radiolabeled ligands.^74^ To date, PRRT has been employed in only 22 cases, covering a variety of tumor subtypes, including somatotroph, lactotroph, somatolactotroph, corticotroph, gonadotroph, hormone-negative, and nonfunctioning tumors, and tumors of unknown subtype.^75^ Among these cases, at least 4 were carcinomas. Among the 19 cases for which information concerning radiological response was available, only 3 showed a partial response, and 5 had stable disease. Interestingly, most of the tumors that demonstrated a partial response or stable disease had not been previously treated with TMZ. In contrast, those tumors previously treated with TMZ either showed disease progression or outcomes were unreported.^6,17,72^ This suggests that PRRT might be particularly effective when employed early in treatment and in combination with certain other treatment modalities, such as radiosensitizers or immunotherapy.^76^ Adverse effects that have been reported include cytopenia, a significant increase in facial pain, and potential pituitary apoplexy.^51,65,75,76^ Ongoing clinical research is exploring several avenues to enhance the effectiveness of PRRT. These include considering earlier implementation of PRRT, investigating new radioligands such as radiolabeled antagonists rather than analogs, using radioligands with different SST subtype affinity profiles, and exploring combinations of PRRT with radiosensitizers or immunotherapy.^77^

### Treatment of Metastasis

Loco-regional treatments of PC metastasis with surgery, RT, or other ablation procedures should be discussed on a case-by-case basis, taking into account the total burden of systemic disease, the performance status of the patient, and the possibility of controlling the primary tumor. It has also to be noted that a dissociated response can be observed with medical treatments.^6,17^ In these cases, the possibility of loco-regional treatment of a single systemic lesion that has not responded can be considered.^78^

## Conclusion

**Figure 1.**
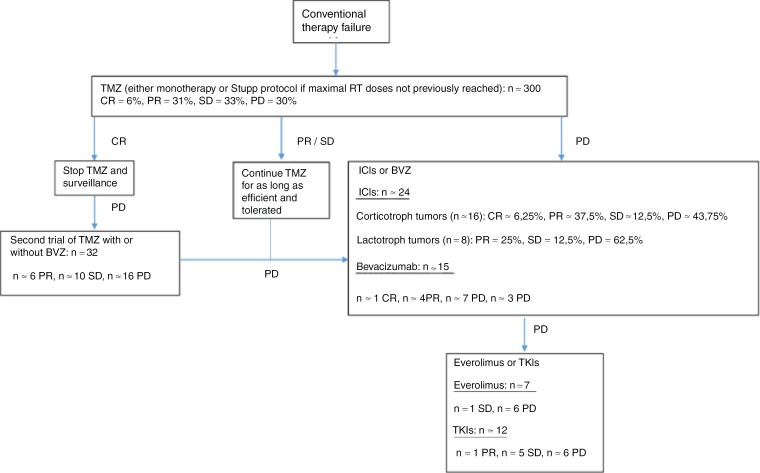
Suggested flowchart for the management of aggressive pituitary tumors and pituitary carcinomas. The most recent results are included. TMZ, Temozolomide; BVZ, Bevacizumab; ICI, Immune-checkpoint inhibitors; TKI, Tyrosine kinase inhibitors; CR, complete response; PR, partial response; SD, stable disease; PD, progressive disease.

APTs and PCs are rare yet challenging conditions requiring a specific multidisciplinary approach in PTCOEs. Their aggressive behavior and potential for metastasis can manifest many years after the primary diagnosis, underscoring the need for vigilant follow-up in select patients. The absence of a clear definition and predictive markers poses challenges in daily clinical practice. If standard therapy fails, TMZ represents the first-line treatment option. PRRT may also be considered, especially in tumors expressing specific SST receptors. When tumors progress after TMZ treatment, the prognosis is typically poor, and among the various second-line treatment options ICIs have proven to be the most effective (Figure 1). Further studies exploring new potential targeted therapies and predictive factors for pituitary tumor aggressiveness are now essential to improve the management and outcomes for these patients.
